# Trait Acclimation Mitigates Mortality Risks of Tropical Canopy Trees under Global Warming

**DOI:** 10.3389/fpls.2016.00607

**Published:** 2016-05-11

**Authors:** Frank Sterck, Niels P. R. Anten, Feike Schieving, Pieter A. Zuidema

**Affiliations:** ^1^Forest Ecology and Forest Management Group, Wageningen UniversityWageningen, Netherlands; ^2^Centre for Crop Systems Analysis, Wageningen UniversityWageningen, Netherlands; ^3^Ecology and Biodiversity Group, Department of Biology, Utrecht UniversityUtrecht, Netherlands

**Keywords:** carbon budget, climate change, functional plant trait, mechanistic plant model, optimization, plasticity, tropical forest, water relations

## Abstract

There is a heated debate about the effect of global change on tropical forests. Many scientists predict large-scale tree mortality while others point to mitigating roles of CO_2_ fertilization and – the notoriously unknown – physiological trait acclimation of trees. In this opinion article we provided a first quantification of the potential of trait acclimation to mitigate the negative effects of warming on tropical canopy tree growth and survival. We applied a physiological tree growth model that incorporates trait acclimation through an optimization approach. Our model estimated the maximum effect of acclimation when trees optimize traits that are strongly plastic on a week to annual time scale (leaf photosynthetic capacity, total leaf area, stem sapwood area) to maximize carbon gain. We simulated tree carbon gain for temperatures (25–35°C) and ambient CO_2_ concentrations (390–800 ppm) predicted for the 21st century. Full trait acclimation increased simulated carbon gain by up to 10–20% and the maximum tolerated temperature by up to 2°C, thus reducing risks of tree death under predicted warming. Functional trait acclimation may thus increase the resilience of tropical trees to warming, but cannot prevent tree death during extremely hot and dry years at current CO_2_ levels. We call for incorporating trait acclimation in field and experimental studies of plant functional traits, and in models that predict responses of tropical forests to climate change.

## Introduction

The effect of climate change on tropical forests is highly uncertain and subject to a heated debate (Körner, 2009; Lewis et al., 2009; Clark et al., 2010; Rammig et al., 2010; Corlett, 2011). One of the prominent concerns is the risk of large-scale tree mortality when trees are gradually pushed outside their current temperature envelop (Wright et al., 2009; Schippers et al., 2015), or confronted with extreme hot and dry years (Phillips et al., 2009; Schippers et al., 2015). Given the importance of tropical forest trees for the global carbon cycle and in heat and water vapor exchange with the atmosphere (Bonan, 2008), large scale mortality of trees may have enormous consequences for global climate and has been identified as one of the tipping points of the whole earth system (Cox et al., 2004).

Several global dynamic vegetation models have predicted the conversion of moist Amazonian forest to seasonal forests or savannah under the warming projected for the coming century (Cox et al., 2004; Malhi et al., 2009). On the other hand, scientists have pointed to two factors that may buffer trees against warming: higher photosynthesis and improved water-use efficiency at high ambient CO_2_ (Lloyd and Farquhar, 2008), and the plastic physiological and morphological responses of trees to climate change (trait acclimation) (Galbraith et al., 2010; Corlett, 2011; Smith and Dukes, 2013). There is ample empirical evidence for increasing photosynthesis at high CO_2_ (Körner, 2009) and there are some indications of increased water-use efficiency (Hietz et al., 2005), but the implications for tree growth and survival are uncertain (Rammig et al., 2010; Corlett, 2011; Zuidema et al., 2013) and subject to debate (Körner, 2009).

In spite of its potential importance in mitigating the negative impacts of warming on tropical forest trees, trait acclimation has so far remained notoriously understudied (Wright et al., 2009; Corlett, 2011). In a recent review on tropical forests and global warming, the importance of acclimation in long-lived canopy trees was emphasized, as “many individual trees alive today will still be living in 2100” (Corlett, 2011), implying that the degree to which tropical trees can acclimate to climate change will critically determine the future of tropical forests. Studies on acclimation responses of tropical trees conducted so far, suggest that they may acclimate to increased temperatures (Way and Oren, 2010) and drought (Metcalfe et al., 2010), and under certain conditions to elevated CO_2_ concentrations (Körner, 2009). Yet, the potential effect of trait acclimation on tree carbon gain under climate change has not been quantified so far (Corlett, 2011) and is not incorporated in current dynamic global vegetation models, DGVMs (Galbraith et al., 2010; Huntingford et al., 2013). If trait acclimation mitigates negative effects of warming for trees and forests, it may increase maximum temperatures at which tropical trees survive with potential implications for the risks of tropical forest dieback.

## A Mechanistic Approach

**FIGURE 1:**
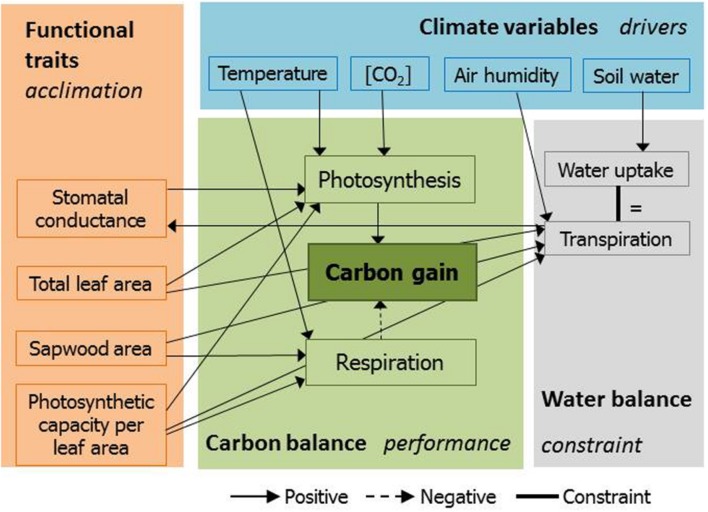
**A simplified representation of the climate impacts on the functioning of trees in the used mechanistic plant model.** Climate-related variables (blue) drive photosynthesis and respiration (green) and determine the water balance (gray). The innovation is the acclimation of functional traits (orange), which is realized by optimizing trait values to maximize carbon gain while maintaining the water balance (see Supporting Information). The water balance component ensures that rates of transpiration, water uptake and water transport are equal.

There is a dire need for a mechanistic approach to projecting tropical tree growth under climate change to quantify the suggested effects of warming, CO_2_ fertilization and trait acclimation (Malhi et al., 2009; Corlett, 2011; Hirota et al., 2011; Cox et al., 2013, Huntingford et al., 2013). Here we show the potential contribution of acclimation in functional plant traits to carbon gain of tropical forest trees. We considered the acclimation of morphological and physiological features that are plastic on time scales of weeks to years (leaf photosynthetic capacity, crown total leaf area, stem sapwood area) and that affect tree growth and survival (Violle et al., 2007). We developed and used a mechanistic, mathematical, plant model that calculates the daily carbon gain of trees based on the hydraulic tree structure accounting for the acquisition, transport and transpiration of water, leaf stomatal coordination, and chemistry and temperature dependency of photosynthesis and respiration of C_3_-plants (**Figure 1**, Supporting Information Text S1; Sterck et al., 2011). We used the model to evaluate the impacts of temperature and ambient CO_2_ on the net carbon gain of rainforest canopy trees. This approach is unique in the sense that it uses optimization theory to quantify the maximum possible contribution of trait acclimation to stimulate carbon gain, tree growth, or mitigate mortality risks under climate change scenarios (Dewar et al., 2009). We simulated trees that optimally acclimate their total leaf area, stem sapwood area and leaf photosynthetic capacity to maximize net carbon gain. Those traits are highly plastic in tropical trees and involve trade-offs because their positive impacts on gross photosynthesis are accompanied by higher respiration costs, higher production costs to replace leaves and sapwood (**Figure 1**, Sterck and Schieving, 2011) and more water loss (**Figure 1**, McMurtrie et al., 2008; Dewar et al., 2009).

We simulated trait acclimation and carbon gain of trees for a wide range of temperatures (25–35°C) and atmospheric [CO_2_] concentrations (390–800 ppm) which are projected for tropical forests in this century (Knutti et al., 2005, IPCC, 2007; Supporting Information Figure S1, IPCC, 2013). We simplified this first exploratory analysis by neglecting more complex diurnal or seasonal patterns in atmospheric conditions, or soil conditions. These sets of simulations allowed us to provide a first bench mark figure for the maximum attainable effect of trait acclimation on tropical canopy trees exposed to gradual warming that occurs along with rising [CO_2_]. In our simulations, non-acclimating trees possess traits optimized at 25°C. We kept relatively humidity constant at 70% which results in an increased transpiration demand at higher temperatures. In our main set of simulations we kept soil water potential at 0 MPa for the entire range of climatic conditions, thus assuming that soil water was not limited. In the Supporting Information Figure S2, we also presented simulations with drier soil conditions (soil water potential of -0.5 MPa).

## Model Structure and Processes

We used a mechanistic plant model (Sterck and Schieving, 2011; Sterck et al., 2011, 2014) to simulate the carbon gain and survival of virtual tropical canopy trees in response to different climate scenarios for ambient CO_2_, temperature and water stress, under the assumption that trees maximize carbon gain by acclimating in their leaf area index, stem sapwood area and leaf photosynthetic capacity. The plant model captures the aboveground structure and physiology of a tree. The modeled trees consist of a cylindrical crown, with a given top height, crown bottom height, crown radius, sapwood area and total leaf area. Leaves are assumed to be uniformly distributed within the crown. The crown is assumed to have an average nitrogen concentration per unit leaf area, and this nitrogen is distributed optimally over the crown in parallel to the light gradient in the canopy following predictions made by big-leaf models (Sellers et al., 1992). Crown photosynthesis is calculated from a biochemical photosynthesis model (Farquhar et al., 1980), a stomatal conductance model and water transport model (Tuzet et al., 2003), temperature dependencies of photosynthetic (protein) and respiratory processes for C_3_-plants in the range of 25–35°C (Bernacchi et al., 2001, 2003), respiratory processes of sapwood for tropical forest trees (Meir and Grace, 2002), and the scaling procedures from leaf to whole tree level (Sterck et al., 2011). A detailed mathematical description of the model is included in Sterck et al. (2011) and in the Supporting Information Text S1. We parameterized the model for a 30 m tall canopy tree exposed to open sky light conditions. Photosynthetic parameters and their temperature responses were fixed for typical C_3_ plant values (Bernacchi et al., 2001, 2003), and mass based sapwood respiration rate, turnover rate of sapwood and leaves, specific sapwood conductivity, and stomatal sensitivity to leaf water potential were set at constant values (Supporting Information Table S1 for complete list of parameter values). The constant trait values reflect a scarcity in quantitative information on acclimation and lack of understanding in underlying trade-offs. Given the potential impact that these traits have on plant responses to climate (Choat et al., 2012; Scoffoni et al., 2012), we call for research on their possible acclimation.

## Modeling Optimization Procedure

We used an optimization framework to determine optimal values for crown leaf area index, stem sapwood area and leaf photosynthetic capacity that maximize carbon gain, and thus mimic the maximum contribution of acclimation in these traits to the carbon gain and survival of trees under climate change. This approach was used to simulate trees under future climate change. We determined the acclimation (i.e., through phenotypic plasticity) of trees to climatic changes such that their net carbon gain is maximized, to set a benchmark for the potential contribution of trait acclimation to future tree performance. This optimization takes the most important constraints on tree carbon gain into account: the co-limitation of photosynthesis by carboxylation and electron transport processes through leaf nitrogen partitioning between these two photosynthetic processes, and steady state for water uptake, transport and loss through coordination of stomatal conductance by tuning the leaf water potential (for details, see Sterck and Schieving, 2011). The traits which we allowed to acclimate are: crown leaf area index, stem sapwood area and leaf photosynthetic capacity (i.e., the average photosynthetic nitrogen mass per leaf area). The values of other functional plant variables were predicted by the implemented processes and include, among others, the crown water potential, intercellular CO_2_ concentration, stomatal conductance, transpiration, nitrogen partitioning between electron transport and carboxylation processes, gross photosynthesis, respiration costs and turnover costs (Supporting Information Table S2 for complete list). Overall, this optimization approach allows us to track emergent multiple trait patterns from underlying principles. Trade-offs occur because increasing values of traits that enhance gross photosynthesis also tend to entail larger respiration costs, as well as higher production costs to replace leaves and sapwood (turnover costs).

## Model Assumptions

The modeling approach is based on a number of assumptions (see Sterck and Schieving, 2011): (i) Leaf nitrogen is optimally partitioned within leaves such that carboxylation and electron transport co-limit photosynthesis. (ii) The crown is characterized by an average leaf water potential ψ_l_ and average intercellular CO_2_ concentration c_i_ inside leaves, calculated over the average vertical and horizontal distance from stem base to leaf in the crown (Sterck et al., 2011). (iii) Leaf temperature equals the air temperature. This approach does not account for vertical gradients in ψ_l_, c_i_ or leaf temperature within the crown, but allows us to scale from leaf to whole tree model and solve that whole tree model for optimized values of traits. (iv) There is steady state for transpiration and stem water flow, which is a reasonable assumption on the 24-h time scale considered here. We assumed that a canopy tree is hydraulically limited in photosynthesis, replaces leaves and sapwood in steady state, and has a 15% surplus of carbon budget left for net vegetative or reproductive growth. We started all simulations from the same set of default parameters (Supporting Information Table S1), but included temperature dependencies of typical C_3_ plants for photosynthetic and respiratory parameters (Sterck et al., 2011, Supporting Information Table S2).

## Potential Acclimation Impacts

**FIGURE 2:**
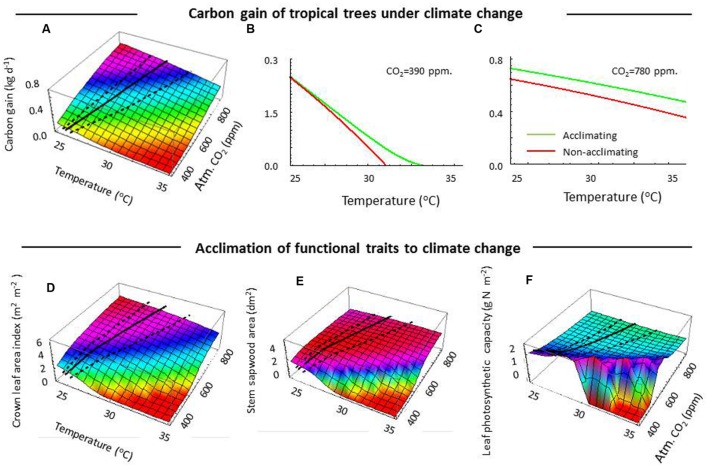
**The simulated responses of tropical rainforest canopy trees to changes in ambient temperature and ambient CO_2_.** **(A)** Net carbon gain in relation to temperature and ambient CO_2_ for an acclimating tree; **(B)** Net carbon gain for acclimating and non-acclimating trees, at recent ambient CO_2_; **(C)** Idem, at twice the ambient CO_2_; **(D)** Crown leaf area index for acclimating trees; **(E)** Stem sapwood area for acclimating trees; **(F)** Leaf photosynthetic capacity, expressed as the average photosynthetic nitrogen mass per unit leaf area. In 3D plots, the black line represents the average predictions of coupled changes in ambient CO_2_ and temperature, dashed lines mark the 95% confidence limits of those changes (Supporting Information Figure S1).

We simulated carbon gain of tropical forest canopy trees in response to different combinations of ambient atmospheric CO_2_ and temperatures. An initial comparison of carbon gain for acclimating and non-acclimating trees reveals higher values for acclimating trees for the full range of temperatures and at two CO_2_ concentrations (**Figures 2B,C**). Yet, at increasing temperatures, acclimating trees could not avoid declines in carbon gain (**Figure 2B**). These results are consistent with empirical observations that growth of tropical forest trees is reduced both during extremely warm and dry years (Feeley et al., 2007; Phillips et al., 2009). The results are consistently found at both current ambient and double ambient CO_2_ level (**Figures 2B,C**), with higher values in the latter case reflecting a potential CO_2_ fertilization effect.

A conspicuous result of our simulations is that at current CO_2_ levels acclimation responses to temperature allowed trees to increase the temperature at which carbon gain approaches zero by an estimated 2°C (**Figures 2A–C**). This result was robust for different scenarios of vapor pressure differences between atmosphere and leaves and different soil water conditions (Supporting Information Figure S2). This result suggests that acclimation will mitigate the effects of warming on changes in the geographical distribution of tropical forests. So far, predictions of the impact of climate change in tree performance and distribution were based on either climate envelops by current distribution patterns (Wright et al., 2009), leaf models (Lloyd and Farquhar, 2008), plant models (McMurtrie et al., 2008) or vegetation models (Sitch et al., 2008). None of these approaches considered the role of acclimation in functional traits such as total leaf area, stem sapwood area and leaf photosynthetic capacity based on biophysical principles, as we did here. Those studies may thus have overestimated the negative impact of warmer and drier conditions on tropical tree distributions. Our prediction that acclimation may extend the climate envelop of tropical forest canopy trees by a maximum of 2°C is particularly relevant at the lower range of ambient CO_2_ levels, and suggest that acclimation increases the resilience of tropical trees to warming predicted for the coming decades.

## Acclimation and Co_2_ Offset Warming Impacts

The predicted decrease in carbon gain with rising temperature was robust over the full range of simulated CO_2_ levels (**Figure 2A**). Similarly, the predicted increase in carbon gain at elevated ambient CO_2_ was robust over the full range of simulated temperatures (**Figure 2A**). Our simulations suggest that the carbon gain of acclimating trees will gradually increase over time, when considering the predicted coupled changes in ambient CO_2_ and temperature (**Figure 2A**, solid line) and their confidence interval (i.e., based on the uncertainties in IPCC scenarios, dashed lines, **Figure 2A**). This suggests that for this century the increasing ambient CO_2_ levels together with trait acclimation will more than offset the negative impacts of warming (**Figure 2A**). This finding is in line with the predicted responses of leaf-level photosynthesis (Lloyd and Farquhar, 2008) and observed elevated photosynthesis and leaf sugar loads under elevated ambient CO_2_ (Körner et al., 2005). When considering tree carbon gain, our results suggest that the negative impacts of temperature and water stress on tree carbon gain will likely be more than offset by the positive impacts of trait acclimation and increase in ambient CO_2_ under the 2–4°C warming scenarios predicted for the end of this century. The predicted resilience of tropical rain forest trees by acclimation and CO_2_ fertilization is consistent with the existence of species rich tropical forests in warm, CO_2_ rich episodes 57 million years ago, when rainfall patterns were not affected (Jaramillo et al., 2010). Yet, the predicted increase in carbon gain needs to be interpreted with care. The doubts about a direct translation of carbon gain into tree (biomass) growth (Körner, 2009), the highly variable biomass responses in CO_2_ enrichment experiments in temperate forests (Körner et al., 2005), the absence of such experiments in the tropics (Zuidema et al., 2013), and isotope/tree ring studies indicating that CO_2_-induced simulation of tree growth may not be valid (van der Sleen et al., 2014) call for caution in relating changes in carbon gain to changes in tree biomass growth.

## Tree Trait Acclimation From Physiological Principles

How can acclimation mitigate the negative effects of warming or enforce the positive effects of higher ambient CO_2_? At constant ambient CO_2_, the simulated trees acclimated to a higher temperature and water stress by decreasing leaf area (**Figure 2D**) and partial closure of stomata (to limit water loss), resulting in reduced leaf photosynthesis. Moreover, a lower leaf area and corresponding lower total nitrogen and protein mass in the crown allowed trees to keep respiration costs (which are strongly determined by protein turnover rates and thus protein content) within the bounds of the photosynthetic carbon supply, i.e., maintaining a positive carbon gain. Trees also maintained a relatively constant stem sapwood area (**Figure 2E**), and the resulting increase in the ratio of sapwood area to leaf area enabled them to maintain a more favorable balance between water supply and transpiration demand, allowing high stomatal conductance and photosynthesis rates at leaf level. The reduction in crown leaf area index resulted in greater light penetration into the canopy and induced a slightly higher average leaf photosynthetic capacity (**Figure 2F**). The predicted acclimation patterns agree with empirical studies showing that tropical trees reduce their leaf area if experimentally subjected to drought (Meir and Woodward, 2010, also for shrub and grasses, Xu and Zhou, 2006; Xu et al., 2014), that trees combine a reduction in leaf area with increased leaf photosynthetic capacity when nitrogen is not limiting (McMurtrie et al., 2008), and that trees establish a lower leaf to sapwood area ratio under drier climatic conditions (Martínez-Vilalta et al., 2009).

**FIGURE 3:**
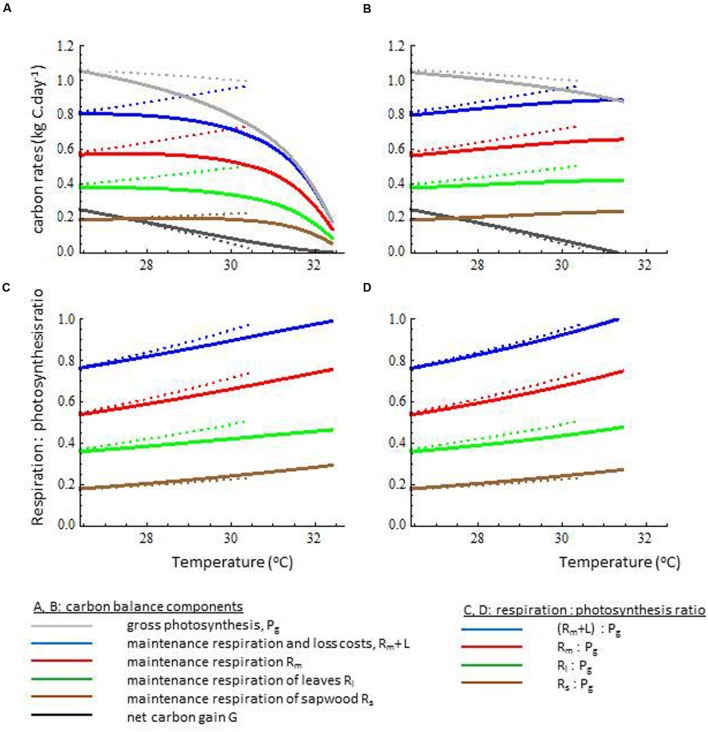
**The impact of acclimation on **(A,B)** the carbon budget components and **(C,D)** the emergent respiration : photosynthesis ratios.** Trees with full acclimation in crown leaf area index, leaf photosynthetic capacity and sapwood area (solid lines in **A** and **C**) were compared with trees with acclimation in leaf photosynthetic capacity and sapwood area but with crown leaf area fixed (solid lines in **B** and **D**), and with non-acclimation trees having fixed values for crown leaf area index, leaf photosynthetic capacity and sapwood area (dotted lines in all sub-figures). For broader interpretation of simulation results, see **Supplementary Figures S3** and **S4**.

**FIGURE 4:**
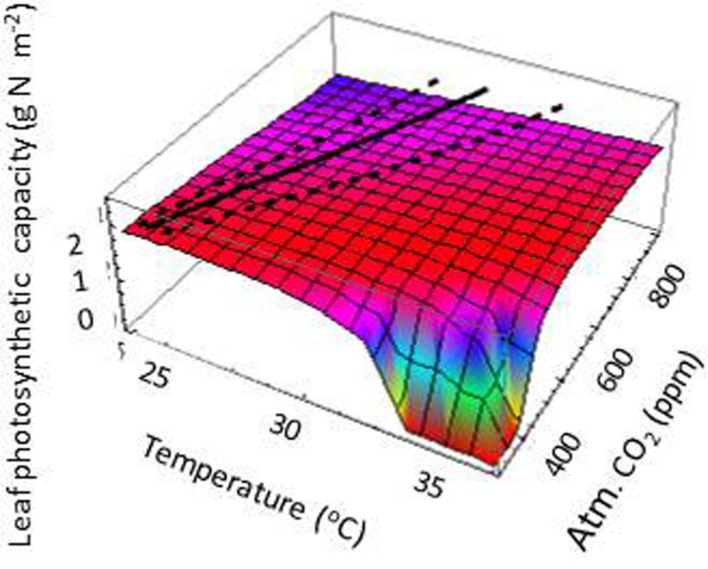
**The acclimation in the leaf photosynthetic capacity when the crown leaf area is kept fixed.** This response may be more relevant for the temperature impacts on leaf acclimation on short time scales (days) than the responses in fully acclimating trees, where the response in LAI (**Figure 2D**) triggers another response in leaf photosynthetic capacity (compare with **Figure 2F**).

Acclimating trees were predicted to maintain a positive carbon gain (i.e., respiration being lower than photosynthetic carbon supply) over a wider temperature range with a ∼ 2°C higher maximum temperature than non-acclimating trees (**Figures 2B,C**). These 2°C higher upper range temperature value was robust for different water stress scenarios, both when differing in the vapor pressure difference between atmosphere and leaves and when considered different levels of soil water availability (Supporting Information Figure S2). Both acclimating and non-acclimating trees were assumed to have the same increase in biochemical respiration costs of photosynthetic proteins under warming (Supporting Information Text S1, Bernacchi et al., 2001, 2003). Such protein-based temperature effects on respiration may account for the observed instantaneous increases in leaf respiration at higher temperature in experimental studies (Bernacchi et al., 2001; Gifford, 2003; Smith and Dukes, 2013; Slot et al., 2014). Our simulations predict that acclimating trees mitigate the additional, temperature-induced, respiration costs at the whole tree level by reducing total leaf area (**Figure 2D**). They thus reduced the total amount of photosynthetic active proteins in the crown, resulting in lower crown maintenance respiration costs (**Figure 3A**). However, on the short run (i.e., days to weeks) trees may not respond in crown leaf area but rather in leaf physiology. When crown leaf area index was kept constant, simulated trees reduced their leaf photosynthetic capacity and leaf respiration (**Figures 3B** and **4**), which is in line with short-term leaf responses to temperature in experimental (Gifford, 2003; Loveys et al., 2003; Smith and Dukes, 2013), modeling studies (Dewar et al., 1999), and field studies on tropical canopy trees and lianas (Slot et al., 2014). At the level of the entire tree, both fully and partially acclimating trees tended to homeostasis in the maintenance respiration: photosynthesis ratio at increasing temperature (**Figures 3C,D**), which is an emergent property of our simulations based on modeled physiological principles and trait acclimation. Our results are also consistent with the empirical long-term respiration responses of plants to warming (Gifford, 2003; Loveys et al., 2003), which are much lower than the often assumed Q_10_ values of 2 (but see Slot et al., 2014), and the slight increase in the ratio for plants at temperatures beyond 23°C (Loveys et al., 2003). Based on our results, we expect that acclimation in crown leaf area index is most relevant for climate impacts over a scale of weeks or more. On a scale of days to weeks, acclimation in the leaf photosynthetic capacity and leaf respiration becomes more relevant (see also Smith and Dukes, 2013; Slot et al., 2014), probably next to substrate limitations for respiration (Dewar et al., 1999) but those were not considered here. Yet, since the mechanisms driving acclimation in tree respiration remain, poorly understood (Teskey et al., 2008), the uncertainty in the understanding and predicting tree growth in response to warming still remains (Smith and Dukes, 2013; Slot et al., 2014).

With increasing ambient CO_2_, our simulated trees produced more leaf area (**Figure 2D**) due to increased photosynthetic efficiency. The higher level of self-shading, a consequence of a larger crown leaf area index, resulted in a lower optimal photosynthetic leaf capacity (**Figure 2F**). These trends in leaf area and photosynthetic capacity with increasing CO_2_ are in line with the results of FACE experiments (Ainsworth and Rogers, 2007) and experiments on small plants (Xu et al., 2014). Since high ambient CO_2_ allows leaves to maintain high leaf photosynthesis but reduce transpiration by a partial closure of stomata, trees increased water-use efficiency (carbon gain/water loss) and decreased sapwood area (**Figure 2E**) to reduce respiratory and replacement costs of sapwood (Ryan et al., 2006). The reduced leaf photosynthetic capacity (**Figure 2F**) at elevated CO_2_ is consistent with observations of lower carboxylation rates (Medlyn et al., 1999; Millard et al., 2007). The increased water-use efficiency at elevated CO_2_ is consistent with results of stable isotope (^13^C) studies in tropical tree rings (Hietz et al., 2005). The predicted reduction in stomatal conductance also agrees with the observation of a 34% decline in stomatal densities for sub-tropical tree species over the last 150 years (Lammertsma et al., 2011). Thus, the predicted acclimation responses are qualitatively supported by empirical studies, and likely contribute to enhancing carbon gain at high ambient CO_2_.

## Extremely Hot and Dry Years

Gradual climatic changes may have very different impacts on tree growth compared to short extreme events, such as the incidental dry and warm years that are affecting tropical forests (Bonan, 2008; Phillips et al., 2009) and will likely increase in frequency due to deforestation and forest fragmentation (Malhi et al., 2008). During such droughts temperatures may increase by as much as 3–5°C (Phillips et al., 2009). We evaluate the effects of droughts in additional simulations in which soil water was drastically reduced. These simulations suggest that during dry and hot years, carbon gain is severely reduced, with high risks of zero or negative carbon gain when such droughts occur under current ambient CO_2_ levels (see Supporting Information Figure S2). Under those conditions, the simulated trees collapsed in total leaf area, stem sapwood area and leaf photosynthetic capacity, similar to the situation at temperatures >30°C, current ambient CO_2_ (390 ppm) and normal soil moisture that is shown in **Figures 2D–F**. Such a collapse confirms the risks for tipping points (Phillips et al., 2010), which is consistent with the increased death rates of large forest trees during warm and dry years (Phillips et al., 2009, 2010). At doubled ambient CO_2_ levels predicted for the end of this century, simulations for dry and hot years showed higher carbon gains and a higher survival probability (Supporting Information Figure S2). Our results thus suggest that tropical trees will become more resilient to individual extreme hot and dry years as ambient CO_2_ increases toward the end of this century. Note, however, that these simulations do not take shifts in rainfall regimes into account, and do not account for extreme case scenarios of warming and drought (Cox et al., 2004).

## Limitations

We based our mathematical model on the recent progress made in physiological leaf models (Bernacchi et al., 2001, 2003; Lloyd and Farquhar, 2008), plant models (McMurtrie et al., 2008; Dewar et al., 2009; Sterck et al., 2011) and vegetation models (Sitch et al., 2008), and added the physiological acclimation in (plastic) functional traits like leaf area, sapwood area and leaf photosynthetic capacity within the constraints set by the biophysical principles of the water and carbon relations within plants (**Figure 1**, Sterck and Schieving, 2011). Our approach provides a step in understanding the role of trait acclimation in mitigating the effects of warming on tropical forest canopy trees, but more work is needed to fully understand this role. Using optimization techniques, our model yields the maximum attainable contribution that the acclimation in some key functional plant traits can make to tree carbon gain and survival. This is the maximum attainable value since, by allowing traits to reach optimal values, possible genetic or time constraints on acclimation are not taken into account. Our simulations also did not consider the possible warming impacts on investments in reproduction, costs of root respiration and maintenance of mycorrhiza, all of which could reduce the realized impact of trait acclimation. On the other hand, we did not include the potentially important drought-induced acclimation in stomatal regulation and cavitation vulnerability, both of which may increase the effect of acclimation. Little is known about the magnitude and the rate at which tree traits respond to increased temperature, drought and elevated ambient CO_2_. Our simulations thus present a first clear benchmark for the maximum attainable impact of acclimation on the climate sensitivity of tropical trees; being 10–20% in terms of tree growth and about 2 C in terms of high-temperature tolerance. The extent to which this potential will be achieved depends on the degree of plasticity in the traits that we considered, but also in traits that were considered not plastic and entered as constants in the simulations. More research on this plasticity is urgently needed.

## Conclusion

Our study suggests that trait acclimation may assist tropical forest trees to survive under the climatic changes predicted for this century. Positive effects of CO_2_ fertilization and trait acclimation on tree carbon gain may mitigate negative impacts of warming and gradually increasing water stress, as long as they remain within the thermal limits of C_3_-photosynthesis of woody plants (Bernacchi et al., 2001, 2003). On the other hand, our simulations reveal that strongly reduced carbon gain and risks of tree death remain during hot and dry years when tree structure and physiology may collapse; tropical forest trees will unlikely be able to adjust to those conditions. These risks are considerable under current and near-future CO_2_ levels, but may be smaller at the doubled CO_2_ concentrations projected for the end of this century (IPCC, 2007, 2013). However, it remains highly uncertain whether acclimation and CO_2_ impacts will be sufficient to mitigate the mortality risk of canopy trees that are exposed to extreme warming (up to ∼9°C) and rainfall loss in largely deforested landscapes (Cox et al., 2004). Under those circumstances, large-scale tree death or crown thinning (Phillips et al., 2009) may provoke further reductions in tree cover, reduce rainfall at regional scale and bring moist forests close to tipping points of conversion to drier forests or even savannahs (Malhi et al., 2009; Hirota et al., 2011). Limiting the magnitude of warming and reducing tropical deforestation during this century will reduce chances that extreme drought events will bring tropical forests close to such tipping points.

We argue that acclimation of functional plant traits and their underlying physiology in tropical canopy trees requires considerably more attention from researchers. We simulated the consequences of optimized acclimation in functional traits that are notoriously plastic and have strong impact on the water balance and carbon gain of trees. The match between the model predictions and observations on acclimation suggests that our approach captured many of the key principles driving the responses of canopy trees to climate change. Our simulations nevertheless present a first benchmark for the attainable impact of acclimation on the climate sensitivity of tropical trees; being 10–20% in terms of tree net productivity (which is rather similar to the predictions of Slot et al., 2014) and about 2°C in terms of high-temperature tolerance. The extent to which this potential will be achieved depends on the degree of plasticity in the traits that we considered, but also in traits that were considered not plastic and entered as constants in the simulations. Even when limited in their acclimation, traits like cavitation vulnerability and stomatal sensitivity to leaf water potential (Sterck et al., 2012) certainly require attention in future studies given their potential impacts on plant responses to climate (Choat et al., 2012; Scoffoni et al., 2012). Such understanding in acclimation is urgently needed to understand how trait acclimation within trees and trait variation across species will drive the resilience of canopy trees that currently dominate the tropical forest, as well as their future replacement by trees of the same or other species. In addition to the rapidly increasing body of literature on trait comparisons across species, better information on acclimation responses is needed to realistically quantify the impact of acclimation on simulated tree growth and forest biomass.

## Author Contributions

All authors contributed to designing this study, drafting and revising the work. FS and FSc developed the plant model used and FS carried out the analysis for this study.

## Conflict of Interest Statement

The authors declare that the research was conducted in the absence of any commercial or financial relationships that could be construed as a potential conflict of interest.
