# Supplementary material for: Trait Acclimation Mitigates Mortality Risks of Tropical Canopy Trees under Global Warming
Source: Front Plant Sci. 2016 May 11;7:607. doi: 10.3389/fpls.2016.00607 (PMC4863428; doi:10.3389/fpls.2016.00607)
Supplement: Supplementary file 1 [file Presentation_1.PDF]

The potential of trait acclimation to mitigate mortality risks of tropical canopy trees under global warming

Frank Sterck, Niels P.R. Anten, Feike Schieving and Pieter A. Zuidema

Supporting information

Text S1. Plant model: description, optimization, parameterization, and scenarios.

(adapted from Sterck et al. 2011)

Plant mass and acclimating traits

The vegetative plant mass *M* is the sum of structural leaf mass M_w, the photosynthetic protein leaf mass M_n, and the sapwood wood mass M_s (all in kg).

$$M = M_w + M_n + M_s \quad \text{[S1]}$$

with,

$$M_w = \rho_w A_l \quad \text{[S2]}$$

$$M_n = \rho_n n_1 A_l \quad \text{[S3]}$$

$$M_s = \rho_s A_s L_s \quad [S4]$$

Where ρ_w is the structural leaf mass per leaf area (kg m^{-2}), ρ_n is the photosynthetic protein mass per nitrogen mass (kg kg^{-1}), ρ_s is the sapwood wood mass per sapwood volume (kg m^{-3}), n_l is the amount of nitrogen per unit leaf mass that is associated with photosynthesis (kg kg^{-1}), A_l is the total leaf area (m^2), A_s is the stem sapwood area below the crown (no tapering) (m^2), and L_s is the average sapwood length between the stem base and leaves (m). These equations thus formalize the impact of the optimized, acclimating variables (A_l, A_s and n_l) on the masses of different components and, in turn, on respiration and loss costs (see next section). For details and for the impact of these variables on whole-plant photosynthesis P_g in particular, we refer to (Sterck et al. 2011, Sterck & Schieving 2011).

Carbon gain

In a previous study (Text S1 in Sterck et al. 2011), carbon gain calculations were based on the net whole plant photosynthesis P_n (kg C d^{-1}) and calculated from the difference between gross photosynthesis P_g and maintenance respiration costs of leaves and sapwood R_m (both in kg C d^{-1}). Here, the carbon costs required to pay for the replacement of leaf and sapwood biomass losses L (kg C d^{-1}) was also accounted for. The net carbon gain G (kg C d^{-1}) was thus calculated as the difference between gross photosynthesis minus the maintenance costs and the replacement costs of leaves and sapwood, all expressed in kg C.d^{-1} (adapted from Sterck & Schieving)

$$G = P_g - R_m - L \quad [S5]$$

The maintenance respiration rate R_m sums the respiration in leaves and sapwood,

$$R_m = r_w M_w + r_n M_n + r_s M_s \qquad [S6]$$

Here r_w and r_s are the parameters for mass based maintenance respiration rates of the structural leaf mass and the sapwood (kg C kg^{-1} day^{-1}), and r_n is the respiration rate per photosynthetic leaf protein mass (kg C kg^{-1}(proteins) day^{-1}). We calculated r_w, r_s and r_n from the parameterized values at a reference temperature of 25°C (r_{w25}, r_{s25}, r_{n25} in Table S1) and temperature responses in those variables over a temperature range from 25-35°C (see next section).

The carbon loss rate L includes the turnover of leaves and sapwood (Sterck and Schieving 2011),

$$L = c_g [\tau_l (M_w + M_n) + \tau_s M_s] \qquad [S7]$$

Here c_g (kg C kg^{-1}) is the parameter for the construction cost per biomass, which includes the carbon mass per unit biomass and the respiratory carbon cost required for the construction of biomass. The τ_l is the leaf turnover rate (day^{-1}) and τ_s is the sapwood turnover rate (day^{-1}).

Here, P_g depends on the light intercepted by the crown leaf area and the leaf photosynthetic capacity (see also Sterck and Schieving 2011). The gross photosynthesis of the plant P_g (kg C day^{-1}) is calculated from,

$$P_g = 43200 \cdot 12 \cdot 10^{-9} \cdot P_g \quad [S8]$$

Here P_g is the gross photosynthetic rate of the plant (μmol C s^{-1}), and the number provides the conversion from (μmol C s^{-1}) to (kg C day^{-1}) when accounting for 12 sunlight hours per day. P_g is calculated from a biochemical photosynthesis model (Farquhar et al. 1980), and formulated as a big leaf model. The gross photosynthesis is described as the minimum of two dependent processes, i.e. carboxylation or rubisco limited photosynthesis rate and electron transport limited photosynthesis rate. Here, the carboxylation limited photosynthetic rate P_c and electron transport limited photosynthetic rate P_j (μmol C s^{-1}) can be written as,

$$P_c = V_{c\max}\{\frac{c_i - \Gamma}{c_i + K_{cmm}\left(1 + {}^{o}\!/_{K_{omm}}\right)}\} \quad [S9]$$

$$P_j = J_{\max}\varphi_j(\xi)\{\frac{c_i - \Gamma}{c_i + 2\Gamma}\} \quad [S10]$$

Where V_{cmax} (μmol C s^{-1}) is the maximum rate of carboxylation of the plant; J_{max} (μmol ^{els} s^{-1}) is the maximum electron transport rate limited photosynthesis; c_i is the effective crown CO_2 pressure across leaves at the focal point (Pa); Γ is a parameter for the CO_2 compensation point (Pa); K_{cmm} (Pa) and K_{omm} (Pa) are the Michaelis Menten constants for carboxylation and oxygenation, respectively; and o is the crown oxygen pressure (Pa) set to the atmospheric O_2 pressure; φ(ξ) is a term for the light limitation effects. V_{cmax} (μmol C s^{-1}) and J_{max} can be written as,

$$V_{c\max} = k_c(1-\upsilon_{chl})(1-\upsilon_j)N_{mass}LMA.LAI.A_c \quad \text{[S11]}$$

$$J_{\max} = \frac{k_j}{4}(1-\upsilon_{chl})\upsilon_j N_{mass}LMA.LAI.A_c \quad \text{[S12]}$$

Here the parameter k_c is the carboxylation capacity per nitrogen mass (μmol C kg^{-1} N s^{-1}); k_j is the electron transport capacity per nitrogen mass (μmol C kg^{-1} N s^{-1}); υ_{chl} is a fixed nitrogen fraction distributed to chloroplasts; υ_j is the nitrogen fraction partitioned to electron transport υ_j. It is assumed that υ_j and the nitrogen fraction partitioned to carboxylation υ_c sum to unity ($\upsilon_j + \upsilon_c = 1$); We thus assumed that all nitrogen is deployed for photosynthesis.

The *φ(ξ)* term in equation is a dimensionless function that specifies the light limitation effects on the electron transport limited photosynthesis. The function *φ(ξ)* varies between 0 and 1, increases linearly from 0 at low values of ξ and approaches 1 asymptotically at high ξ values,

$$\varphi(\xi) = \frac{(1+\xi) - \sqrt{(1+\xi)^2 - 4\theta_j \xi}}{2\theta_j} \qquad [S13]$$

with θ_j the curvature factor for the non-rectangular hyperbola and ξ is the ratio of the light absorption rate over the capacity for the electron transport (dimensionless), defined as,

$$\xi = \frac{qI_{aF}}{k_j (1-\upsilon_{chl})\upsilon_j . N_{mass} . LMA . LAI} \qquad [S14]$$

Where q is the quantum yield (μmol electrons μmol photons^{-1}) and I_{aF} is the absorbed light per unit ground area (μmol photons m^{-2} s^{-1}) calculated as,

$$I_{aF} = I(1 - Exp[-K_l LAI]) \qquad [S15]$$

Here I is the vertical light intensity on top of the crown (μmol photons m^{-2} s^{-1}), and K_l is the light extinction coefficient (dimensionless) for leaves.

We can formulate c_i as a function of the effective crown water potential ψ_l. We assumed steady state for the CO_2 influx and the CO_2 consumption by the crown,

$$G_s \frac{(c_a - c_i)}{p_a} = P_n \qquad \text{[S16]}$$

Here G_s is the stomatal conductance of the plant (μmol C s^{-1}); P_n is the net crown photosynthesis rate of the plant (μmol C s^{-1}); c_a is atmospheric CO_2 pressure (Pa); and p_a is the atmospheric pressure set at 1.10^5 Pa. G_s is modelled by scaling of a function for leaf stomatal conductance (Tuzet et al. 2003) to the whole crown,

$$G_s = G_{s0} + a \frac{P_n}{c_i - \Gamma} g_{\psi} \qquad \text{[S17]}$$

Here G_{s0} is the residual stomatal conductance of the plant (μmol C s^{-1}) and the parameter a is a scaling parameter set to the value 2 p_a. A logistic equation describes a dimensionless stomatal sensitivity g_{ψ} to ψ_l (Tuzet et al. 2003),

$$g_{\psi}(\psi_l) = \frac{1 + e^{a\psi \times \psi ref}}{1 + e^{a\psi \times (\psi ref - \psi l)}} \qquad \text{[S18]}$$

which for the given parameter values a_{ψ} and ψ_{ref} varies from 0 ($\psi_l << \psi_{ref}$) to 1 (at ψ_l=0 MPa). Since we assumed that G_{s0}=0, P_n cancels from the CO_2 balance equation, and the calculation of c_i is simplified to an equation of ψ_l and a number of parameters,

$$c_i(\psi_l) = \frac{(a/p_a)g_\psi(\psi_l)c_a + \Gamma}{(a/p_a)g_\psi(\psi_l) + 1} \quad \text{[S19]}$$

Steady state is assumed for the plant transpiration E and the plant water transport through the sapwood F. While not realistic at a time scale of minutes to hours, such a steady state is a reasonable assumption for tree life and size patterns in the daily time step model used here. We can write this steady state assumption for the transpiration and stem water flow, i.e. E –F = 0,

$$\gamma_{wc} a \frac{P_n(\psi_l)}{c_i(\psi_l) - \Gamma} g_\psi(\psi_l) D - (\psi_b - \psi_g - \psi_l)\frac{K_s A_s}{L_s} = 0 \quad \text{[S20]}$$

For the left term, γ_{wc} is the ratio of water diffusivity over carbon dioxide diffusivity (~1.6) and D the vapor pressure difference between leaf and air. The right term is the product between the pressure difference between stem base and focal point in the crown and the average sapwood conductance between the stem base and a focal point in the crown (represented by the mean vertical and horiztonal distance to a leaf in the crown). The presssure difference is based on the stem base pressure ψ_b, crown potential pressure ψ_l and the presssure loss due to gravity ψ_g. Sapwood conductance is written as the product of the specific conductivity of sapwood K_s, the sapwood area A_s divided by the sapwood length L_s between stem base and leaf in the crown.

temperature dependent variables

The temperature dependencies of leaf photosynthetic and respiratory parameters were added, using the generic temperature response equation,

$$parameter = \exp(c - \Delta H_{\alpha} / RT_{K}) / c_{n} \qquad [S21]$$

R is the molar gas constant (0.00831 kJ K^{-1}), T_K is the leaf temperature (in K), c is a scaling constant and ΔH_α is the activation energy (in KJ mol^{-1}), respectively (Bernacchi et al. 2001; 2003). The parameter values for carboxylation capacity k_c and electron transport capacity k_j were photosynthetic protein based while assuming fixed nitrogen mass fractions in those proteins in our model (see Text S1, S2 in Sterck et al. 2011, but not in the model of Bernacchi et al. 2001; 2003).We added a scaling term c_n to normalize the parameter values for the Michaelis-Menten constants for carboxylation K_{cmm} and oxygenation K_{comm} to 1 at 25°C (not done in (Bernacchi et al. 2001). Based on multiple experiments, it was shown that the trends in c and ΔH_α were robust across different C_3 plant species over a range of temperatures (10-40° C, Bernacchi et al. 2001; 2003). We used the parameter values for c and ΔH_α as provided by Bernacchi et al. (2001; 2003) to estimate the temperature dependencies in the 25-35°C range. In our simulations, the normalized parameter values were multiplied with the parameter values at 25°C (e.g., k_j=k_{j25} ×*parameter*).

Table S1.1. The parameterization of the Arrhenius equation [S21] for the temperature responses of the nitrogen (protein) based carboxylation capacity k_c, the nitrogen (protein) based electron transport capacity k_j, the Michaelis-Menten constant for carboxylation K_{cmm},

the Michaelis-Menten constant for oxygenation K_{omm}, and the photosynthetic nitrogen (protein) based leaf respiration r_n and structural leaf mass based respiration r_w. Q_{10} values show the corresponding relative increases in respiration over the 25-35°C range.

Parameter (see also Table S1)	code	c	ΔH_α	c_n	Q_{10}
Carboxylation capacity per unit nitrogen	k_c	26.35	65.33	1	2.35
Electron tranport capacity per unit nitrogen	k_j	17.7	43.9	1	1.78
Michaelis Menten constant for carboxylation	K_{cmm}	38.05	79.43	404.9	2.83
Michaelis Menten constant for oxygenation	K_{omm}	20.30	36.38	278.4	1.61
Mass-based respiration rate for photosynthetic leaf mass	r_n	18.72	46.39	1	1.84
Mass-based respiration rate for structural leaf mass	r_w	18.72	46.39	1	1.84

These settings reflect the protein based temperature responses in photosynthetic parameters, and for leaf respiration. For simplicity, we parameterized the respiration of nitrogen mass in structural leaf material r_w in the same way as r_n. In all simulations, the respiration rate of the structural leaf mass ($r_w \times M_w$) was inferior to the respiration of the photosynthetic leaf (protein) mass ($r_n \times M_n$) in the crown.

Information on temperature-dependency of sapwood maintenance respiration is scarce. A study of *in situ* measurements of woody tissue respiration of two tropical forests (Meir and Grace 2002) suggests that the temperature responses in r_s can be described with Q_{10} values ranging from 1.6 (± 0.1 SE) to 1.8 (± 0.1 SE). In line with this, we calculated r_s from:

$$r_s = r_{s25} Q_{10s}^{(T-25)/10} \quad [S22]$$

Where r_{s25} refers to the mass based respiration rate of sapwood at 25°C, Q_{10s} to the increase in this respiration rate over a range of 10 degrees C, and T to the actual temperature (in ^{o}C). We used a Q_{10s} of 1.7 (average value from Meir and Grace, 2002) to simulate the temperature response in r_s. Thus we assumed respiration per unit of photosynthetic nitrogen (or protein) mass and structural mass in the leaves and per unit sapwood mass, to be exponentially related to temperature. This does however not entail a similarly exponential relationship at the whole-plant level. As discussed in the main text, our model accounts for longer-term acclimation to temperature as our optimization procedure allows for acclimation in total leaf area A_l, leaf photosynthetic capacity n_l and sapwood area A_s. As a result our whole-plant respiration responses to temperature are comparable in shape to the long-term responses elsewhere (e.g., Gifford 1995, 2003, Dewar et al. 1999, Atkins et al. 2005).

Optimization

A key assumption of our analyses is the ability of plants to optimally acclimate (i.e., through phenotypic plasticity) to climatic changes such that their net carbon gain (*G* in Eq. S5) is maximized. The traits which we allowed to acclimate are: crown leaf area index, stem sapwood area and leaf photosynthetic capacity (i.e. the average photosynthetic nitrogen mass per leaf area). Values of these traits were adjusted as to maximize *G* (Eq. S5). This optimization takes the most important constraints on tree carbon gain into account: the co-limitation of photosynthesis by carboxylation and electron transport processes through leaf

nitrogen partitioning between these two photosynthetic processes, and the steady state for water uptake, transport and loss through coordination of stomatal conductance by tuning the leaf water potential (for details, see Sterck and Schieving 2011). Trade-offs occur because higher values of optimized traits increase both benefits in terms the gross photosynthesis P_g, and the costs in terms of respiration R_m and turnover L (see Fig. 1 in main text). Because of its complexity, the objective function G was solved numerically using the Nelder Mead algorithm in the Mathematica software package 8.0 (Mathematica 2008).

Parameterization

The sources and values used for the parameterization are provided by Table S1. Below, we shortly explain this parameterization (partially adapted from Text S2 in Sterck et al. 2011). We considered averaged diurnal environmental conditions, because this allows us to evaluate carbon gain rate patterns in a simplified way. The external vertical light intensity I was set at an arbitrary level of 1000 μmol m^{-2} s^{-1} for 12 daylight hours, and at 0 μmol m^{-2} s^{-1} for the remaining 12 night hours (reasonable values for the tropics). The atmospheric air pressure p_a=100000 Pa. The other environmental variables (temperature, soil water potential, atmospheric vapour pressure and ambient CO_2 were set for a range of values to explore the possible consequences of climate change (see also Methods). We simulated trees in a temperature range from 25-35°C and an atmospheric CO_2 concentration range from 390-800 ppm. Moreover, we carried out this procedure for three water stress scenarios: an optimistic (low water stress) scenario characterized by a vapour pressure difference D of 950 Pa (which corresponds with a relative air humidity of 70% at 25°C) between atmosphere and leaves (even with increasing temperature) and saturating soil water conditions (soil water potential ψ_b=0 MPa); an intermediate scenario that differs from the optimistic one because D is

calculated from the temperature and a constant relative humidity of 70%; and a pessimistic scenario that differs from the intermediate one by drier soil water conditions (ψ_b = -0.5 MPa).

Most photosynthetic parameters are relatively stable across C_3-plants (Bernacchi et al. 2001; 2003, Lambers et al. 1998) and were standardized at 25°C (Tables S1). We estimated these standardized values at 25°C (Sterck et al. 2011) for the carboxylation capacity per unit nitrogen k_{c25}=83000 μmol (kg N)$^{-1}$ s^{-1} and the electron transport capacity per unit nitrogen k_{j25}=1050000 μmol (kg N)$^{-1}$ s^{-1} (Sterck et al. 2011, Sterck & Schieving 2011); the Michaelis Menten constant for carboxylation K_{cmm25}=40.4 Pa and for oxygenation K_{omm25}=24800 Pa; the oxygen concentration in the leaf o=21000 Pa; the quantum yield q=0.25 (μmol electrons). (photon^{-1}); the ratio for H_2O/CO_2 diffusivity γ_{wc}=1.6; the curvature factor for the electron transport rate process θ_j=0.5. Other rather stable parameter values included the fraction of incident light absorbed by a leaf K_l=0.86 (Lambers et al. 1998); and the protein mass per unit photosynthetic nitrogen mass ρ_{ns}=5.88 was estimated (Sterck & Schieving 2011).

Mass based respiration rate parameters were estimated. The reported estimates for the sapwood mass based respiration rate vary a lot in the literature and are prone to error (Teskey et al. 2008). Based on previous simulations (Sterck & Schieving 2011), we estimated the sapwood mass based respiration rate r_{s25} of 0.4 μmol C kg^{-1} s^{-1}. For leaves, the structural leaf maintenance respiration rate r_{w25} was set at 4 μmol C kg leaf s^{-1} (Sterck & Schieving 2011). For the photosynthetic respiration rate, a maintenance respiration rate per unit nitrogen r_{n25}=1000 μmol C kg^{-1} (protein) s^{-1} was assumed (Wright et al. 2005; Reich et al. 2008; Sterck et al. 2011, Sterck & Schieving 2011).

Here k_{c25}, k_{j25}, k_{cmm25}, k_{omm25}, r_{s25}, r_{n25} and r_{w25} represent the reference respiration rates at 25°C (Table S1). The corresponding respiration rates k_c, k_j, k_{cmm}, k_{omm}, r_s, r_n and r_w (Table S2) between 25-35°C were calculated as the product of a normalized temperature dependency (Eq. S21) and the reference respiration rates at 25°C.

We set the leaf mass per leaf area *LMA* at 0.05 kg m^{-2} (Lambers et al. 1998); specific hydraulic conductivity K_s at 1.5 kg m^{-1} s^{-1} MPa^{-1} (Maherali et al. 2004); the leaf water potential at which plants lost half of their stomatal conductance capacity ψ_{ref} at -1.9 MPa (Tuzet et al. 2003); and the sapwood mass density ρ_s at 500 kg m^{-3} (Maherali et al. 2004).

We parameterized the model for a 30 m tall tree (h_t=30 m) to represent an " average" adult canopy tree of a tropical forest. We set the crown radius *r* at 5 m and the crown bottom height h_b at 20 m, which is the vertical distance from the soil to the lowest leaf. The crown depth was thus assumed to be 10 m. We optimized leaf area density (leaf area per crown volume) λ_l (m^2 m^{-3}), sapwood area density (sapwood area per crown volume) λ_s (m^2 m^{-3}), and leaf photosynthetic nitrogen mass per leaf area n_l (g N m^{-2} leaf) at a temperature of 25° C and ambient CO_2=390 ppm. From the leaf area density and sapwood area density, we calculated the crown leaf area index (in m^2 m^{-2}, λ_l /10, since crown depth is 10 m) and stem sapwood area (in dm^2, from $\lambda_s \times V_c \times 10^2$, with a cylindrical crown volume $V_c = 10\ \pi\ 5^2 = 785\ m^3$). We interpret this tree as an adult canopy tree at maximum size. Assuming steady state for leaf and sapwood production in such a tree and an investment of 15% (G/P_g=0.15) of the carbon gain into reproduction (Green & Johnson 1994, Thomas 1996), we were able to set the sapwood mass based maintenance rate r_{s25} and turnover rate τ_s as the closing entries of the carbon budget (Sterck & Schieving 2011). All parameterized trait values are listed in Table S1 and the calculated variables in Table S2.

Climate change and water availability scenarios

We simulated all possible combinations for a range of atmospheric CO_2 pressures (390-800 ppm) and temperatures (25-35° C), and highlighted the simulation results for an average scenario (with 95% confidence intervals) of coupled changes in CO_2 and temperature predicted for this and the coming century (IPCC 2007, Fig S1).

In this supplementary information file, we also show the consequences of the interactive effects between the CO_2-temperature scenarios in pessimistic water availability scenario (as opposed to the intermediate scenario shown in the main text). While the intermediate scenario assumed soil water potential of 0 MPa, this was changed to -0.5 MPa in the pessimistic scenario. This value is within the range of observed values for drought periods (Becker et al. 1988). Figure S2 shows that this scenario yielded qualitatively comparable relations of carbon gain with temperature and ambient CO_2 pressure, but with lower carbon gains and a lower tolerance to high temperature. Under both water availability scenarios, acclimating trees responded to warming by increasing the sapwood- to crown area ratio, and thus by improving the balance between water supply (through sapwood) and demand (through leaf transpiration). In this way, acclimating trees had a smaller reduction in stomatal conductance, crown water potential and internal crown CO_2 concentration with an increasing temperature compared to non-acclimating trees. Leaf photosynthetic capacity (i.e., driven by the average nitrogen mass per leaf area) initially increased with temperature because the average light levels on leaves were higher (due to lower crown leaf area index). With such average higher light conditions, relatively more nitrogen was partitioned from electron transport processes to carboxylation processes to maintain the assumed co-limitation between these processes.

Beyond a certain temperature limit, the crown leaf area index, stem sapwood area and leaf photosynthetic capacity however dropped in parallel to a low level, and this drop was quite dramatic in both the water scenarios. This coupled drop implies that, beyond certain temperature, acclimating trees collapsed in structure and functioning. This happens, for example, when the temperature rises beyond 29°C in the pessimistic scenario under ambient CO_2 of 400 ppm. At that point, the costs of maintaining a 30 m tall structure started to exceed the benefits, even in these optimized, acclimating trees.

Under higher atmospheric CO_2, the critical temperature provoking this tree collapse increased, which is expected since acclimating trees tended to partially close their stomata in response to CO_2 elevation thus reducing water loss. Moreover, trees were predicted to use light more effectively at higher atmospheric CO_2 pressures. This increased light use efficiency was caused because the limitations of carboxylation were relaxed, and acclimating plants allocated more nitrogen to the electron transport process and leaves achieved a more positive carbon balance under shaded conditions. Consequently, the optimally acclimating trees are predicted to increase their crown leaf area index at higher atmospheric CO_2 pressures. A greater crown leaf area index resulted in lower averaged light levels on leaves and thus in a lower optimal leaf photosynthetic capacity. Sapwood tended to decrease with higher ambient CO_2 levels, also because acclimating trees reduced water loss by partial stomatal closure while maintaining high CO_2 intake owing to a greater ambient CO_2 to internal CO_2 concentration difference.

Figure S1: Scenarios for atmospheric CO_2 and temperature

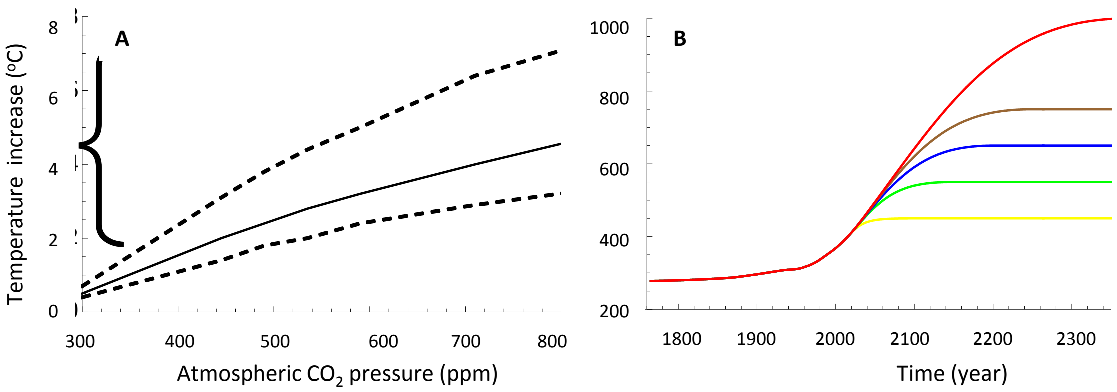

(A) In our simulations, we coupled the increase in atmospheric CO_2 directly to that in temperature. Since this coupling is uncertain, we present the averaged coupling between increasing atmospheric CO_2 and globally increasing temperature (solid lines), and we also do for 90% confidence intervals (dashed lines). Data source: IPCC (2007), Technical summary: Barker et al. 2007; Table TS 2 (p. 39); Figure TS 11 (p. 42). (B) Atmospheric CO_2 concentrations are predicted to increase over the coming century, but may stabilize after 100-200 years from now (Knutti et al. 2005). The trajectories are highly uncertain, because anthropogenic CO_2 emissions are difficult to predict over longer time spans, and the buffering capacity of for example oceans is uncertain. Nevertheless, long-term trajectories have been established for CO_2 scenarios (Knutti et al. 2005, IPCC 2007) and our model considers an ample and likely range in CO_2 and temperatures predicted for this and the coming century.

Figure S2. Different water stress scenarios.

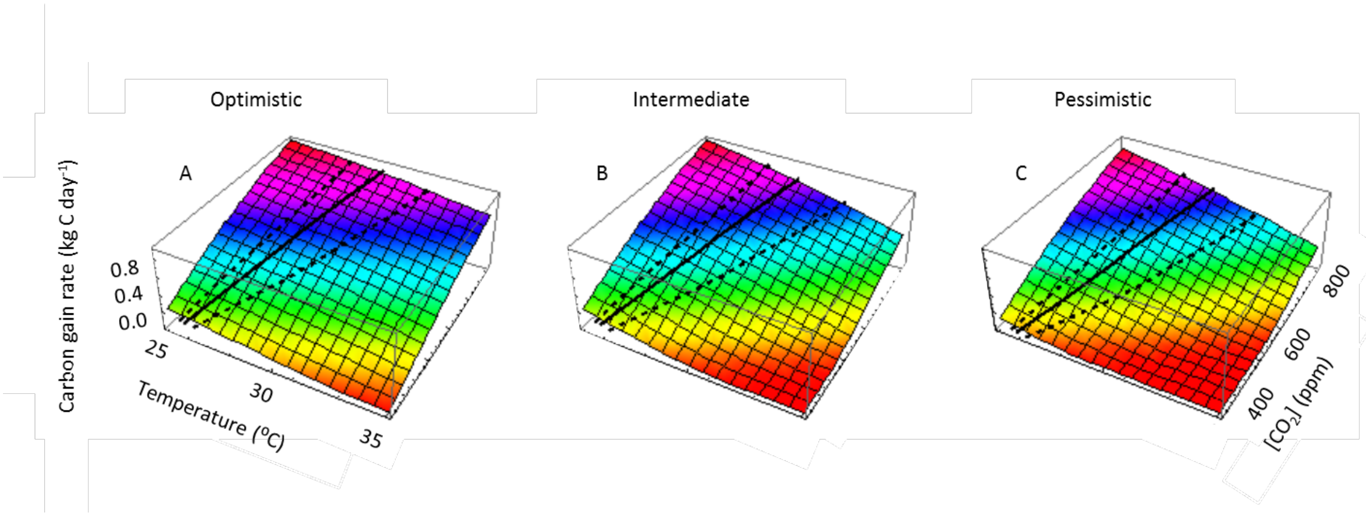

Carbon gain in response to atmospheric CO_2 and temperature for (A) an optimistic, (B) an intermediate and (C) a pessimistic water stress scenario, where the intermediate scenario is presented in the main text. The optimistic scenario (low water stress) is characterized by a vapour pressure difference *D* of 950 Pa between atmosphere and leaves (even with increasing temperature) and saturating soil water conditions (soil water potential ψ_b=0 MPa). The D of 950 Pa corresponds with a relative humidity of 70% at 25°C. The intermediate (water stress) scenario differs from the optimistic one because *D* is calculated from the temperature and a constant relative humidity of 70%. The pessimistic scenario (high water stress) differs from the intermediate one by drier soil water conditions (ψ_b = -0.5 MPa). The results of the intermediate water stress scenario are shown in the accompanying main text.

Figure S3. Acclimation in the carbon budget in response to warming

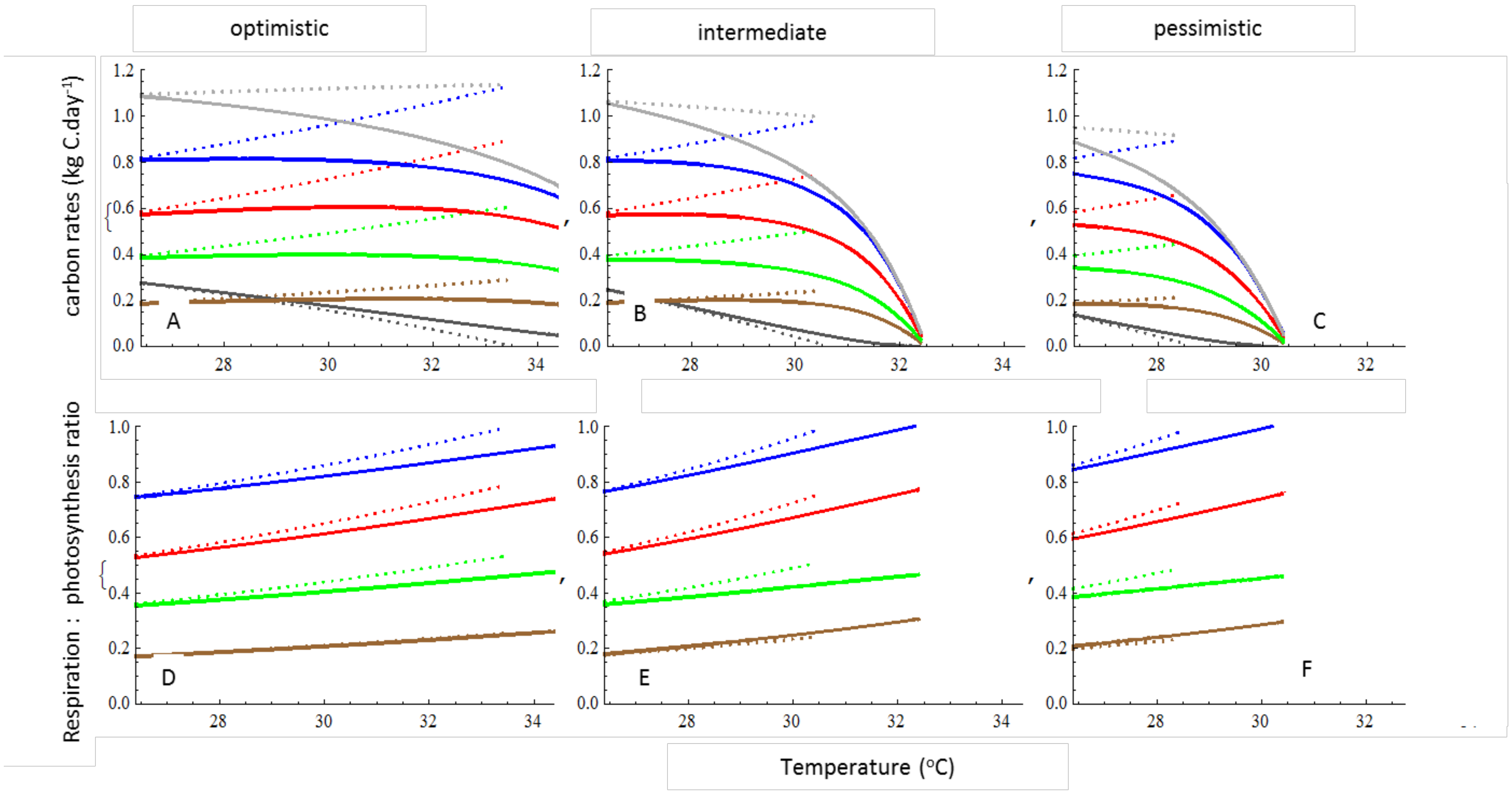

Legend Figure S3A, B, C: carbon balance components

- gross photosynthesis, P_g
- maintenance respiration and loss costs, R_m+ L
- maintenance respiration R_m
- maintenance respiration of leaves R_l
- maintenance respiration of sapwood R_s
- net carbon gain G

Legend Figure S3D, E, F: respiration cost: photosynthesis ratio

- $(R_m+L) : P_g$
- $R_m : P_g$
- $R_l : P_g$
- $R_s : P_g$

Figure S3. The effect of warming on the carbon budget components differed strongly between the acclimating (solid lines) and non-acclimating trees (dotted lines). While acclimating trees adjusted crown leaf area index, stem sapwood area and leaf photosynthetic capacity to maximize carbon gain, the trait values for non-acclimating trees were fixed at the optimized values for 25°C. In these simulations, we kept ambient CO_2 concentration constant at 390 ppm.

Non-acclimating trees (dotted lines in A,B,C): Non-acclimating trees exhibited only a very minor increase in gross photosynthesis under warming, for the optimistic water stress scenario (grey dotted line in Fig. S3 A, see also Fig. S4 A), in which they faced a constant transpiration demand. The trend in gross photosynthesis is explained by the positive impacts of photosynthetic capacity on gross photosynthesis, which more than offset the negative impacts of K_{cmm} on gross photosynthesis. When higher temperatures were accompanied by stronger atmospheric water stress (Fig. S3 B, intermediate scenario) and lower soil water availability (Fig. S3 C, pessimist scenario), gross photosynthesis dropped due to stomatal closure, which mitigated water loss but resulted in low internal leaf CO_2 concentrations and decreasing gross photosynthesis (grey dotted lines) and carbon gain (black dotted lines) with increasing temperature. In non-acclimating trees, the total respiration of sapwood (brown dotted lines), leaves (green dotted lines) and their combined total, increased with temperature (red dotted lines, Fig. S3, A, B, C). Since non-acclimating trees had constant turnover rates and constant replacements costs, the sum of the overall maintenance respiration costs and loss costs (blue dotted lines) showed a similar increasing trend with the increasing temperature (Fig S3, A, B, C).

Acclimating trees (solid lines): The acclimating trees exhibited a much steeper decrease in gross photosynthesis with increasing temperature, compared to non-acclimating trees. This difference was consistent across all three water stress scenarios (grey solid lines in Fig S3, A,B,C). These trees responded to warming by producing fewer leaves, resulting in a lower crown leaf area index (Fig 2D). This acclimation response improved the balance between water supply through the stem and loss through leaf transpiration and thus enabled these plants to maintain conductive stomata and higher internal CO_2 concentrations than non-acclimating plants. This loss in crown leaf area index not only resulted in a lower gross photosynthesis but also in lower maintenance respiration costs of the leaves (green solid lines) and whole tree (red solid lines) and replacement costs (added to maintenance respiration costs, blue lines), thus favouring a more positive carbon balance (the solid black lines). As a consequence, acclimating trees were able to maintain a positive carbon balance at up to 2°C higher temperatures than non-acclimating trees across all water stress scenarios (see the solid versus dotted black lines, also in Fig S2).

There was a considerably smaller increase in ratio between maintenance respiration and gross photosynthesis in acclimating trees than in non-acclimating trees (solid green and red lines vs. dotted lines of the same colour, Fig S3 D,E,F). This notable difference can be explained as follows. Both acclimating and non-acclimating trees experience similar temperature-induced stimulation of protein-based respiration rates in leaves, but in acclimating trees the effect for the whole-plant respiration is mitigated by a reduction in total leaf area. This mitigating effect occurred in spite of the fact that acclimating trees had somewhat larger sapwood respiration (brown lines, Fig. S3, D,E,F), because the relative increase in sapwood area is much smaller than the reduction in leaf area, and also because respiratory costs per unit sapwood mass are smaller than those per unit leaf mass.

While the strong short term responses in leaf respiration to temperature observed in many experiments (Bernacchi et al. 2001; 2003, Gifford 1995, 2003, Loveys et al. 2003, Atkins et al. 2005) may be driven by the temperature impacts on leaf protein- maintenance (Dewar et al. 1999), our results are consistent with the observed long-term acclimation in respiration, as expressed by a tendency to homeostasis in total leaf maintenance respiration to photosynthesis ratio observed for many plant species (Gifford 1995, 2003, Loveys et al. 2003, Atkins et al. 2005). Our results show that trees that acclimate in functional traits for net carbon gain maximization will thus strongly mitigate the response in the maintenance respiration to photosynthesis ratio but may not achieve full homeostasis for this ratio.

Figure S4. Acclimation (but with fixed value for LAI) in the carbon budget in response to warming

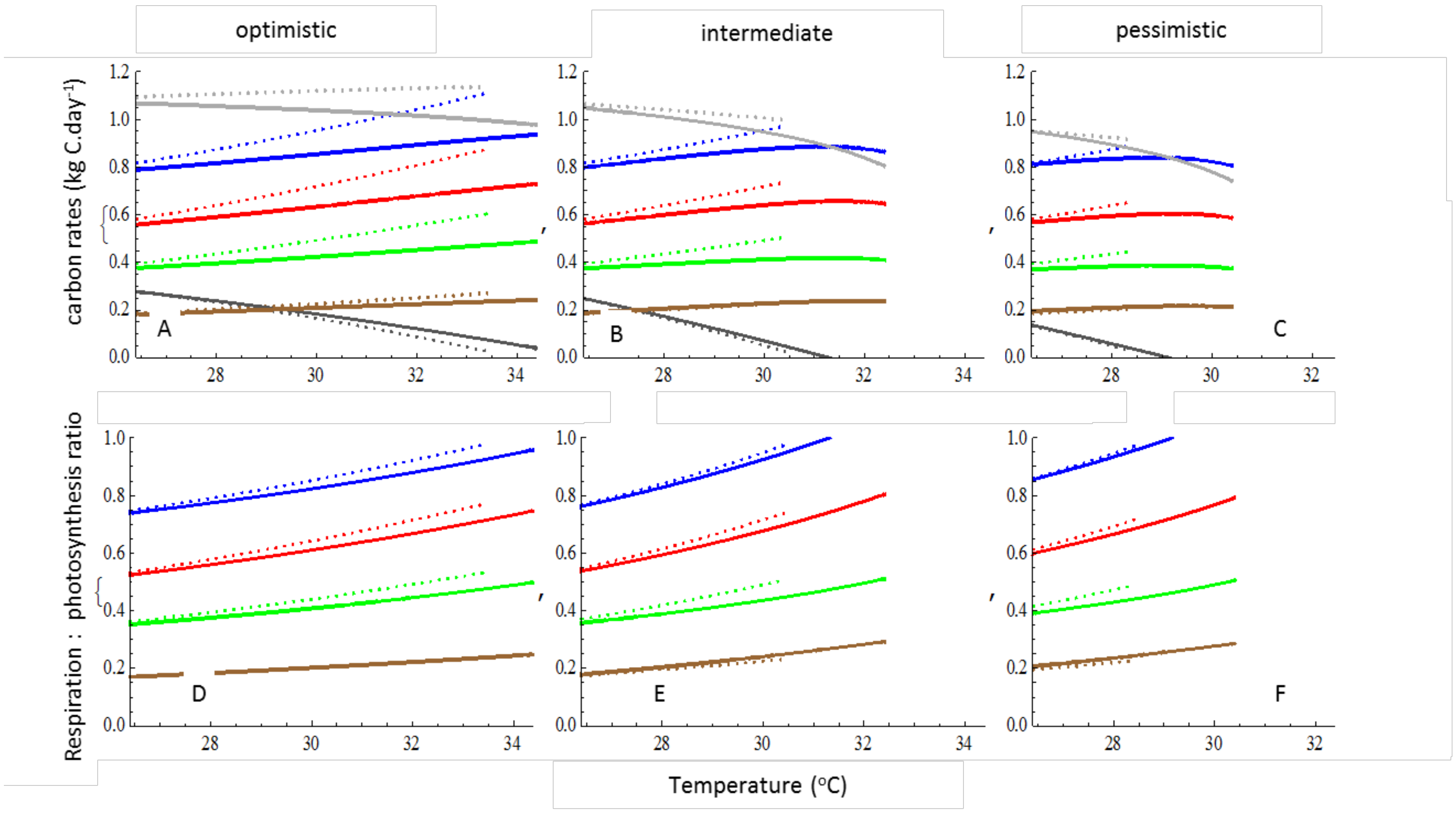

Legend Figure 4A, B, C: carbon balance components

- gross photosynthesis, P_g
- maintenance respiration and loss costs, R_m+ L
- maintenance respiration R_m
- maintenance respiration of leaves R_l
- maintenance respiration of sapwood R_s
- net carbon gain G

Legend Figure S4D, E, F: respiration cost: photosynthesis ratio

- $(R_m+L) : P_g$
- $R_m : P_g$
- $R_l : P_g$
- $R_s : P_g$

Figure S4 presents the same analysis as Figure 3S, but now crown LAI is kept constant to simulate the short term temperature effects on acclimation in leaf photosynthetic capacity. While acclimation in leaf area index occurs on a scale of weeks, the acclimation in the leaf photosynthetic capacity may occur on a scale of hours or days. For this reason, the measured short term responses in leaf photosynthesis and respiration to increasing temperature as is observed in experimental studies is better reflected by simulations that allow for acclimation in leaf photosynthetic capacity while keeping the leaf area index kept constant.

In Figure S4 (A-C), we show the net carbon gain responses for those trees to a range of temperatures and ambient CO_2 for three water stress scenarios (compare with trees with full acclimation, in Fig.S3). Trees without acclimation in the leaf area index achieved lower net carbon gain rates than trees with full acclimation, and they ended up with negative carbon balances at too high temperatures because the higher leaf respiration and turnover costs, owing to a fixed leaf area index, could not be balanced by the gross photosynthesis. Overall, the trees extended the temperature envelop where they are predicted to survive (i.e. maintain positive carbon balance) with ~1°C compare to trees without acclimation (Figure S4, A-C), whereas trees with the full acclimation extended that maximum temperature by ~2°C (Figure S3, A-C). Leaf level acclimation thus only partially compensates for a lack in crown level acclimation in LAI.

With increasing temperature, the trees reduced their leaf photosynthetic capacity (Figure 4, main text) and thus mitigated the change in the photosynthetic : respiration ratio compared to trees without any acclimation (Figure S4, D-F). The leaf level responses are in line with experimental studies, that show such mitigating acclimation responses for a wide range of species over a time scale of hours to days (e.g. Dewar et al. 1999, Gifford 2003, Loveys et al.

2003). Trees with full acclimation, however, decreased their leaf area index so much that their leaf photosynthetic capacity slightly increased with an increasing temperature (Figure 2F, main text). The ratio of the crown photosynthesis to the respiration ratio was thus mitigated in trees with and without leaf area index acclimation, as can be seen by comparing acclimating trees with trees without any acclimation (Figure S4, D-F).

References for Supplementary text and figures

Atkins OK, Bruhn D, Tjoelker MG (2005) Responses of plant respiration to changes in temperature: mechanisms and consequences of variations in Q_{10} values and acclimation. *Plant respiration*, eds Lambers H, Ribas-Carbo M (Springer, the Netherlands), pp 95-135.

Becker P, Rabenold PE, Idol JR, Smith AP. (1988) Water potential gradients for gaps and slopes in a Panamanian moist forest's dry season. *Journal of Tropical Ecology*, **4**, 173-188.

Bernacchi CJ, Pimentel C, Long SP. (2003) In vivo temperature response functions of parameters required to model RuBP-limited photosynthesis. *Plant, Cell and Environment*, **26**, 1419-1430.

Bernacchi CJ, Singsaas EL, Pimentel C, *et al*. (2001) Improved temperature response functions for models of Rubisco-limited photosynthesis. *Plant, Cell and Environment*, **24**, 253-259 (2001).

Dewar RC, Medlyn BE, McMurtrie RE. (1999) Acclimation of the respiration/photosynthesis ratio to temperature: insights from a model. *Global Change Biology*, **5**, 615-622.

Farquhar GD, von Caemmerer S, Berry JA. (1980) A biochemical model of photosynthetic CO_2 assimilation in leaves of C_3 species. *Planta*, **149**, 78-90.

Gifford GM (1995) Whole plant respiration and photosynthesis of wheat under increased CO_2 concentration and temperature: long-term vs. short-term distinctions for modelling. *Global Change Biology*, **1**, 385-396.

Gifford GM. (2003) Plant respiration in productivity models: conceptualisation, representation and issues for global terrestrial carbon-cycle research. *Functional Plant Biology*, **30**, 171-186.

Greene DF, Johnson EA (1994) Estimating the mean annual seed production of trees. *Ecology* **75(3)**, 642-647.

IPCC (2007 *Climate Change 2007: The physical science basis. Contribution of working group I to the fourth assessment report of the intergovernmental panel on climate change*, eds Solomon S, Qin D, Manning M, Chen Z, Marquis M, Averyt KB, Tignor M, Miller HL. (Cambridge University Press, Cambridge UK and NY), 996 pp.

Knutti R, Joos F, Müller, SA, Plattner GK, Stocker TF (2005) Probabilistic climate change projections for CO_2 stabilization profiles. *Geophysical Research Letters,* **32**, 1-4.

Lambers H, Chapin III FS, Pons TL. (1998) *Plant physiological ecology*. Springer, New York.

Loveys BR, Atkinson LJ, Sherlock DJ, Roberts RL, Fitter AH, Atkin OK (2003) Thermal acclimation of leaf and root respiration: an investigation comparing inherently fast- and slow-growing plant species. *Global Change Biol* 9: 895-910.

Maherali J, Pockman WT, Jackson RB. (2004) Adaptive variation in the vulnerability of woody plants to xylem cavitation. *Ecology,* **85(8)**, 2184-2199.

Mathematica (2008) *Wolfram Mathematica Tutorial Collection*, pp. 3668.

Meir P, Grace J. (2002) Scaling relationships for woody tissue respiration in two tropical forests. *Plant, Cell and Environment,* **25**, 963-973.

Reich, PB, Tjoeker MG, Pregitzer KS, *et al*. (2008) Scaling of respiration to nitrogen in leaves, stems and roots of higher lands plants. *Ecology Letters,* **11**, 793-801.

Sterck F, Markesteijn L, Schieving F, &Poorter L. (2011) Functional traits determine trade-offs and niches in a tropical forest community. *Proceedings of the National Academy of Sciences,* **108**(51), 20627-20632.

Sterck F, Schieving F. (2011) Modelling functional trait acclimation for trees of different height in a forest light gradient: emergent patterns driven by carbon gain maximization. *Tree Physiology,* **31**, 1024-1037.

Teskey R, Saveyn A, Steppe K, McGuire MA (2008) Origin, fate and significance of CO_2 in tree stems. *New Phytologist,* **177**, 17-32.

Thomas, SC (1996) Relative size at onset of maturity in rain forest trees: a comparative analysis of 37 Malaysian species. *Oikos,* **76**, 145-154.

Tuzet A, Perrier A, Leuning R. (2003) A coupled model of stomatal conductance, photosynthesis and transpiration. *Plant, Cell and Environment,* **26**, 1097-1116.

Wright IJ, Reich PB, Atkin OK, *et al*. (2005) Irradiance, temperature and rainfall influence leaf dark respiration in woody plants: evidence from comparisons across 20 sites. *New Phytologist,* **169**, 309-319.

Table S1. List of model parameters ("constants") and variables in the model: symbols, units, explanation, input values and literature sources (adapted from Tables S1 and S3 in (1)).

Symbol	Units	Explanation	Input	Ref.
**Roman**				
A	Pa	Parameter for stomatal sensitivity function g_ψ	2	1,2
a_ψ	MPa^{-1}	Slope parameter in stomatal sensitivity function g_ψ	3.2	1,2
c_a	Pa	Atmospheric CO_2 pressure	39[!]	3
D	Pa	Vapor pressure difference between leaves and atmosphere	950[!]	3
G_{s0}	μmol C..s^{-1}	Residual stomatal conductance of the plant	0	
I	$\mu mol.m^{-2}. s^{-1}$	Light intensity in horizontal plane above plant	1000	
h_t	m.	Height of the top of the plant	30	
h_b	m.	Height of the crown bottom	20	
k_{c25}	$\mu mol\ C.kg^{-1}N.s^{-1}$	Carboxylation capacity per unit nitrogen at 25°C	83000	4,5
K_{cmm25}	Pa	Michaelis-Menten constant for carboxylation at 25°C	40.4	6
k_{j25}	$\mu mol\ C.kg^{-1}N.s^{-1}$	Electron tranport capacity per unit nitrogen at 25°C	1050000	4,5
K_{omm25}	Pa	Michaelis-Menten constant for oxygenation at 25°C	24800	6
K_l	-	Light extinction coefficient of crown	0.86	6
o	Pa	O_2 concentration in leaf, same as in atmosphere	21000	6
K_s	$kg.m^{-1}.s^{-1}.MPa^{-1}$	Specific hydraulic conductivity of sapwood	1.5	7
LMA	kg m^{-2}	Leaf mass per leaf area	0.05	6

p_a	Pa	Atmospheric pressure	100000	3
q	μmol. μmol^{-1}	Quantum yield (μmol electrons per photon)	0.25	6
R	m.	Radius of the crown cylinder	5	
r_{n25}	μmol C.kg^{-1} N.s^{-1}	Respiration rate per photosynthetic leaf nitrogen mass at 25°C	1000	1,5
r_{s25}	μmol C. kg^{-1}.s^{-1}	Mass-based respiration rates for sapwood at 25°C	0	1,5
r_{w25}	μmol C. kg^{-1}.s^{-1}	Mass-based respiration rates for structural leaf mass at 25°C	4.0	1,5
**Q_{10s}**	-	Mass-based sapwood respiration increase a 10°C increase	1.7	8
**Greek**				
γ_{wc}		ratio H_2O/CO_2 diffusivity	1.6	4
θ_j	-	Curvature factor for the electron transport rate process	0.5	6
ρ_s	kg.m^{-3}	Sapwood mass per sapwood volume	500	7
ρ_w	kg.m^{-2}	Structural leaf mass per unit leaf area	0.05	6
ρ_n	kg protein. kg^{-1}N	Protein mass per nitrogen mass	5.88	5
Γ	Pa	CO_2 compensation point	3.7	2
τ_l	day^{-1}	Leaf turnover rate	0.05	5
τ_s	y^{-1}	Sapwood turnover rate	1/(6.7×365)	5
ψ_b	MPa	Water potential at the stem basis	0[!]	
ψ_{ref}	MPa	Crown water potential with stomatal sensitivity halved	-1.9	2

[!] baseline values, but a range of values was used in most of our simulations (see Methods, Text S1).

Table S2. The variables that are calculated by the model and based on the optimization of λ_l, λ_s, n_l and υ_j, and used for explanation of the model. Adapted from (1).

symbol	Units	Explanation
**Roman**		
A_l	m^2	Total leaf area
A_s	dm^2	Sapwood cross-section area
c_i	Pa	Internal leaf CO_2 pressure
E	$\mu mol.s^{-1}$, $\mu mol.m^{-2}.s^{-1}$	Transpiration rate of plant.
F	$\mu mol.s^{-1}$	Water flux through plant
G_s	$\mu mol\ C.m^{-2}.s^{-1}$	Stomatal conductance of the plant
I_a	$\mu mol.m^{-2}\ .s^{-1}$	Photon absorption rate by plant
k_c	$\mu mol\ C.kg\text{-}1N.s^{-1}$	Carboxylation capacity per unit nitrogen
K_{cmm}	Pa	Michaelis-Menten constant for carboxylation
k_j	$\mu mol\ C.kg\text{-}1N.s^{-1}$	Electron tranport capacity per unit nitrogen
K_{omm}	Pa	Michaelis-Menten constant for oxygenation
L_s	m.	Average distance between stem base and crown leaves
M_w	kg.	Structural leaf mass
M_n	kg.	Photosynthetic mass in leaves (proteins)
M_s	Kg	Living sapwood mass in plant
M	Kg	Total vegetative plant mass

n_l	kg N m^{-2} leaf	Average photosynthetic nitrogen mass per unit leaf area
P_c	μmol m^{-2} s^{-1}	Carboxylation limited photosynthetis rate of plant
P_g	kg C day^{-1}	Gross photosynthesis rate of the plant per day
P_j	μmol m^{-2} s^{-1}	Electron transport limited photosynthesis rate of the plant
P_n	kg C day^{-1}	Net photosynthesis rate of the plant per day: P_g-R_m
R_m	kg C day^{-1}	Maintenance respiration rate
r_n	μmol C kg protein N^{-1} s^{-1}	Respiration rate per leaf nitrogen mass in photosynthetic proteins
r_s	μmol C kg^{-1} s^{-1}	Mass-based respiration rates of sapwood
r_w	μmol C. $kg^{-1}.s^{-1}$	Mass-based respiration rates for structural leaf mass
V_c	m^3	Crown volume
**Greek**		
λ_l	$m^2.m^{-3}$	Leaf area density in the crown cylinder
λ_s	$m^2.m^{-3}$	Sapwood area density in the crown cylinder
υ_j	-	Fractions of nitrogen allocated to electron transport
ψ_l	MPa	Water potential at the focal point in crown
ψ_g	MPa	Water potential due to gravity in crown cylinder

References for Tables S1 and S2.

1. Sterck F, Markesteijn L, Schieving F, Poorter L (2011) Functional traits determine trade-offs and niches in a tropical forest community. *Proc Nat Ac Sci* 108(51): 20627-20632.
2. Tuzet A, Perrier A, Leuning R (2003) A coupled model of stomatal conductance, photosynthesis, and transpiration. *Plant Cell Environ* 26: 1097-1116
3. Campbell GS, Norman JM (1998) Introduction to Environmental Physics (Springer, New York)
4. Wullschleger SD (1993) Biochemical limitations to carbon assimilation in C_3 plants – a retrospective analysis of A/C_i curves from 109 species. *J Exp Bot* 44: 907-920.
5. Sterck F, Schieving F (2011) Modelling functional trait acclimation for trees of different height in a forest light gradient: emergent patterns driven by carbon gain maximization. *Tree Physiol* 31:1024-1037.
6. Lambers H, Chapin FS, III, Pons TL (1998) *Plant Physiol Ecol* (Springer, New York)
7. Maherali H, Pockman WT, Jackson RB (2004) Adaptive variation in the vulnerability of woody plants to xylem cavitation. *Ecology* 85(8): 2184-2199.
8. Meir P, Grace J (2002) Scaling relationships for woody tissue respiration in two tropical rain forests. *Plant Cell Environ* 25: 963-973.
